# Cynomolgus monkeys are successfully and persistently infected with hepatitis E virus genotype 3 (HEV-3) after long-term immunosuppressive therapy

**DOI:** 10.1371/journal.pone.0174070

**Published:** 2017-03-22

**Authors:** Noemi Rovaris Gardinali, Juliana Rodrigues Guimarães, Juliana Gil Melgaço, Yohan Britto Kevorkian, Fernanda de Oliveira Bottino, Yasmine Rangel Vieira, Aline Campos de Azevedo da Silva, Douglas Pereira Pinto, Laís Bastos da Fonseca, Leandro Schiavo Vilhena, Edilson Uiechi, Maria Cristina Carlan da Silva, Julio Moran, Renato Sérgio Marchevsky, Oswaldo Gonçalves Cruz, Rodrigo Alejandro Arellano Otonel, Amauri Alcindo Alfieri, Jaqueline Mendes de Oliveira, Ana Maria Coimbra Gaspar, Marcelo Alves Pinto

**Affiliations:** 1 Laboratório de Desenvolvimento Tecnológico em Virologia, Oswaldo Cruz, Instituto Oswaldo Cruz, Fundação Oswaldo Cruz, Rio de Janeiro, Brazil; 2 Serviço de Equivalência e Farmacocinética –SEFAR, Vice-Presidência de Produção e Inovação em Saúde–VPPIS, Fundação Oswaldo Cruz, Rio de Janeiro, Brazil; 3 Libbs Indústria Farmacêutica, Embu, São Paulo, Brazil; 4 Laboratório de Biologia Molecular de Patógenos (Virologia Molecular), Centro de Ciências Naturais e Humanas-CCNH, Universidade Federal do ABC-UFABC, São Bernardo do Campo, São Paulo, Brazil; 5 Dr. Julio Moran Laboratories, Ebmatingen, Zurich, Switzerland; 6 Laboratório de Neurovirulência, Instituto de Tecnologia em Imunobiológicos Bio-Manguinhos, Fundação Oswaldo Cruz, Rio de Janeiro, Brazil; 7 Programa de Computação Científica, Fundação Oswaldo Cruz, Rio de Janeiro, Brazil; 8 Laboratório de Virologia Animal, Departamento de Medicina Veterinária Preventiva Universidade Estadual de Londrina, Paraná, Brazil; CEA, FRANCE

## Abstract

Epidemiological studies found that hepatitis E virus genotype 3 (HEV-3) infection was associated with chronic hepatitis and cirrhosis in immunocompromised patients. Our study aimed to investigate the relationship between the host immunosuppressive status and the occurrence of HEV-related chronic hepatitis. Here we describe a successful experimental study, using cynomolgus monkeys previously treated with tacrolimus, a potent calcineurin inhibitor immunosuppressant, and infected with a Brazilian HEV-3 strain isolated from naturally infected pigs. HEV infected monkeys were followed up during 160 days post infection (dpi) by clinical signs; virological, biochemical and haematological parameters; and liver histopathology. The tacrolimus blood levels were monitored throughout the experiment. Immunosuppression was confirmed by clinical and laboratorial findings, such as: moderate weight loss, alopecia, and herpes virus opportunistic infection. In this study, chronic HEV infection was characterized by the mild increase of liver enzymes serum levels; persistent RNA viremia and viral faecal shedding; and liver histopathology. Three out of four immunosuppressed monkeys showed recurrent HEV RNA detection in liver samples, evident hepatocellular ballooning degeneration, mild to severe macro and microvesicular steatosis (zone 1), scattered hepatocellular apoptosis, and lobular focal inflammation. At 69 dpi, liver biopsies of all infected monkeys revealed evident ballooning degeneration (zone 3), discrete hepatocellular apoptosis, and at most mild portal and intra-acinar focal inflammation. At 160 dpi, the three chronically HEV infected monkeys showed microscopic features (piecemeal necrosis) corresponding to chronic hepatitis in absence of fibrosis and cirrhosis in liver parenchyma. Within 4-months follow up, the tacrolimus-immunosuppressed cynomolgus monkeys infected with a Brazilian swine HEV-3 strain exhibited more severe hepatic lesions progressing to chronic hepatitis without liver fibrosis, similarly as shown in tacrolimus-immunosuppressed solid organ transplant (SOT) recipients. The cause-effect relationship between HEV infection and tacrolimus treatment was confirmed in this experiment.

## Introduction

Hepatitis E virus (HEV) infection is the major aetiology of acute viral hepatitis worldwide (http://www.who.int/mediacentre/factsheets/fs280/en/). According to the current taxonomic classification, HEV is classified into the *Hepeviridae* Family, which is divided in two genera: *Orthohepevirus* with four species (A-D) that infect mammals and birds; and *Piscihepevirus* with a single species (A) identified in trouts (http://www.ictvonline.org/virusTaxonomy.asp). The *Orthohepevirus A* species includes the four major mammalian genotypes of human interest: genotypes 1 and 2 (HEV-1 and HEV-2) that infect only humans, and cause large waterborne epidemics in hyperendemic areas; and genotypes 3 and 4 (HEV-3 and HEV-4) that cause autochthonous infections in developing and developed countries and can infect not only humans but also a variety of animal species, such as pigs and other domestic and wild animals [1]. Pigs represent the major reservoir for HEV-3 and HEV-4, which are transmitted by the consumption of raw or uncooked pig meat [2].

Intriguingly, the epidemiologic scenario of hepatitis E in Brazil seems to be closer to that observed in developed countries, where few human cases have been reported. Moreover, in the best of our knowledge HEV-3 is the single genotype circulating in Brazil. [3–5]. HEV-3 is widely disseminated among Brazilian pig herds, and has been detected in pig faeces and effluent of slaughterhouses, as well as in swine livestock products [6–8]. Although HEV infection is largely disseminated among pig herds from different regions, the source of HEV exposure in Brazil remains unclear. It is possible that consumption of raw or undercooked contaminated meat/sausage and exposure to animal hosts may be sources of infection [9]. Other foods like shellfish, vegetables and fruits can be contaminated with HEV and are possible sources of foodborne HEV transmission [10–12].

Both, HEV-3 (more frequently) and HEV-4 infection can persist and become chronic in immunosuppressed patients, mainly in solid organ transplant (SOT) receptors [13–15]. Likewise, patients with haematological disease or coinfected with human immunodeficiency virus (HIV) and low TCD4+ count (<200/mm^3^) can become persistently infected by HEV [16, 17]. Chronic HEV infection is defined by persistent HEV replication for more than three months [18], that can evolve to chronic hepatitis and fibrosis progression quite rapid, within the first two years of infection [18, 19]. Approximately 60% of HEV infected SOT receptors may become chronically infected [19, 20].

Different types of immunosuppressants can modulate viral infection by inhibiting host immunity and/or directly affecting the virus life cycle. Tacrolimus is a potent macrolide immunosuppressant derived from *Streptomyces tsukubaensis* (calcineurin pathway inhibitor) and the most common medication employed to reduce the rate of rejection, especially in parenchymal organ transplantation [21]. The use of tacrolimus is the most important risk factor associated with chronic hepatitis in SOT recipients infected with HEV-3 [19]. High doses of tacrolimus showed to promote infection of liver cells with HEV in cell culture models [22].

Non-human primates (NHP), including *Macaca fascicularis* (cynomolgus monkeys) and *Macaca mulatta* (rhesus monkeys), are primary models for studying the clinical course of HEV infection. NHP are often the most appropriate model to evaluate the zoonotic potential of HEV. Rhesus monkeys are able to mimic a chronic infection by HEV-4 [23], and cynomolgus monkeys are used as animal model for studying pathogenesis of acute HEV infection, which is characterized by liver enzyme elevation, viremia, and seroconversion [24–26]. Besides, cynomolgus monkeys are considered the ideal model for human organ transplantation [27, 28], and have been used for the preclinical development of a broad variety of immunosuppressive agents, including tacrolimus [28, 29]. The present study aimed to investigate the relationship between the host immunosuppressive status of cynomolgus tacrolimus-immunosuppressed and the occurrence of HEV-related chronic hepatitis.

## Methods

### Animals and ethics statement

Twelve clinically healthy cynomolgus monkeys (*Macaca fascicularis*), six males and six females, aged between 1–8 years old, weight from 1.3 to 3.6 kg (Table 1), were used in this study. Animals were obtained from a breeding colony from the Institute of Science and Technology in Biomodels (ICTB), of the Oswaldo Cruz Foundation (Fiocruz), Rio de Janeiro, Brazil at Animal Biohazard Level 2 facilities during quarantine and throughout the whole experiment. Animals were singly housed in stainless steel squeeze-back cages (0.77 m height x 0.60 m width x 0.68 m depth) in a climate-controlled room (temperature of 22 ± 1°C and humidity 55 ± 5%) with a 12h light/dark cycle and fed daily with a commercial primate diet supplemented with fresh fruits and vegetables. Water was provided *ad libitum*. The study protocol was approved (LW-17/13) by the Institutional Animal Care and Use Committee (CEUA-Fiocruz), and conducted in strict accordance with the recommendations from the Guide for Care and Use of Laboratory Animals of the Brazilian Society of Science in Laboratory Animals (SBCAL) and the National Council for the Control of Animal Experimentation (CONCEA, Brazil). The single housing cynomolgus monkey approach was adopted in our study in order to prevent cross contamination, since HEV is transmitted via the fecal-oral route. The housing standard adopted in our study attended to the space recommendations for individually non-human primates with a maximum weight of 7 kg, in accordance with the Brazilian Normative Resolution CONCEA n.28, of November 13, 2015 (http://www.mct.gov.br/upd_blob/0240/240230.pdf). Environmental enrichment programs were offered throughout the study in the form of alimentary (popcorn and nuts), audio-visual (movies and audios with forest themes) and tactile enrichment (toys such as hanging balls). Clinical procedures were performed under anaesthesia, and all efforts were made to minimize painful procedures.

**Table 1:** Gender, age, sex, and body weight of the cynomolgus monkeys used in this study.

Monkey ID	Gender*	Age (yr)	Weight (kg)
AB17	M	4,8	3,66
AD7	M	2,4	2,24
Z2	F	6,8	2,75
AE2	F	1,11	1,62
AD4	F	2,4	1,85
AE3	M	1,5	1,55
V12	F	8,4	3,1
AC11	M	3,4	3,07
AE6	F	1,2	1,3
AB19	M	4,11	3,5
AD8	F	2,3	1,83
AC7	M	3,6	2,94

* M = male; F = female.

### Study design

Monkeys were divided into three groups of four animals, matched by age, weight, and gender. Group 1 (G1) consisted of immunocompetent monkeys (AD4, AE3, V12 and AC11) that were intravenously inoculated with HEV-3. The Group 2 (G2) monkeys (AE6, AB19, AD8 and AC7) were treated with the immunosuppressive tacrolimus and then intravenously inoculated. The Group 3 (G3) animals (AB17, AD7, Z2 and AE2) were immunosuppressed but did not receive the inoculum (negative control). G2 and G3 monkeys started receiving tacrolimus treatment 42 days prior to inoculation. Aiming to evaluate the possibility of HEV infection reactivation, monkeys from G1 (not treated prior to inoculation) started receiving the immunosuppressive treatment from day 91 to 149post-inoculation. All animals, which were from colony free of simian immunodeficiency virus (SIV) and simian type D retrovirus (SRV/D), tested also negative for anti-HEV IgG and IgM and HEV RNA (in sera, faeces, and liver biopsies). Cage side observation of unanesthetized monkeys for signs of illness was performed daily during quarantine and throughout 160 days post infection (dpi). Physical examinations (weight, rectal temperature and body inspection) were performed on all blood collection days under anaesthesia, since the animals were not trained for such procedures. Haematological, biochemical, and serological basal parameters were established for each animal by using three serially obtained individual pre-inoculation samples. Whole blood and faecal samples were collected at 0, 7, 14, 28, 42, 57, 69, 99, 141 and 160 dpi for virological, serological, biochemical and haematological determinations, and for tacrolimus plasmatic concentration. Liver biopsies for virological and histopathological analyses were obtained at 0, 14, 42, 69, 99, 141 and 160 dpi and collected by ultrasound-guided biopsy in monkeys under surgical anaesthesia. For local analgesia, 0.1–0.2 ml of 2% lidocaine was subcutaneously injected at the biopsy site in order to reduce the post-operatory pain at the recovery phase of general anaesthesia. In dorsal decubitus position, the hair trichotomy was performed from the right cranial abdomen and skin disinfection was conducted. A transabdominal ultrasound was conducted to identify an appropriate liver biopsy site free of great vessels, gallbladder, and adjacent organs. Samples were taken from the right medial or right lateral lobe. A sterile Menghine needle (n.16) was slowly advanced under the skin until it was visualized at the appropriate biopsy site and then the spring-loaded biopsy apparatus was discharged to obtain a core of the liver tissue (∼4–8 mg). At the end of the procedure, ultrasound visualization of the biopsy site was maintained to check for signs of haemorrhaging. In addition, bile, liquor and different types of tissues including gallbladder, duodenum, jejunum, ileum, colon, spleen, mesenteric lymph nodes, pancreas and brain were collected from all animals at necropsy. The anaesthetic protocol used in all collection dates was performed with ketamine hydrochloride at 20 mg/kg (Vetanarcol, König, Argentina) and xylazine hydrochloride at 0.1 mg/kg (Syntec Brazil, São Paulo, Brazil). At 160 dpi, animals were euthanized under deep barbiturate anaesthesia with sodium thiopental 2.5% at 25 mg/kg (Thiopentax, Cristalia, São Paulo, Brazil), which was delivered intravenously. Subsequently, animals were submitted to cardiac punctures and then euthanized by exsanguination.

### Tacrolimus treatment

Tacrolimus treatment was based on the therapeutic dose used in cynomolgus monkeys models for human organ transplantation [28]. Forty-two days prior to inoculation, G2 and G3 monkeys were treated orally with 3.5 mg/kg/day of tacrolimus until day 71 pi, and with 2.0 mg/kg/day up to 149 dpi. The immunocompetent HEV infected G1 monkeys started receiving tacrolimus treatment (PO. 2.0 mg/kg/day) from 91 to 149 dpi. The tacrolimus medicine used in this study was kindly donated by Libbs Farmacêutica Ltda/Farmanguinhos/Fiocruz.

### Inoculum

The HEV strain used for inoculation was recovered from faeces of a commercial farmed pig located in Paraná state, Brazil and characterized as genotype 3 (GenBank accession numbers: ORF1 KX578263, ORF2 KX578267). The faecal sample was diluted in phosphate-buffered saline (PBS) (pH 7.4) to make a 10% water/volume (wt/vol) suspension. Subsequently, the clarified suspension was filtered through 0.45 μm and 0.22 μm filters. The inoculum contained a load of 6.4 log_10_ copies per millilitre (ml), as determined by quantitative reverse transcription polymerase chain reaction (qRT-PCR) [30].

### Serological assays

Pre and post-inoculation sera samples (obtained at 0, 14, 28, 56, 99, 141 and 160 dpi) were tested for detection of macaque anti-HEV IgG, IgM and IgA by using the commercially available DiaCheck anti-human HEV antibody assay supplemented with a goat anti-macaque immunoglobulin conjugate (Fitzgerald Industries International, Inc., Massachusetts, USA), according to a modified protocol (Dr. Julio Moran Laboratories, Zurich, Switzerland). The cut off was calculated for each animal as the mean of absorbances of negative samples plus three times the standard deviation of negative samples.

### qRT-PCR

Total RNA from 200 microliter (μl) of serum/liquor and 10% faecal/bile suspensions was purified with the High Pure RNA Isolation Kit (Roche Applied Science, Mannheim, Germany), and from 30 mg of liver and other tissue samples with the RNeasy Mini Kit (Qiagen, Hilden, Germany), according to the manufacturer’s instructions. Five μl of RNA were reverse-transcribed and amplified by using the “AgPath-ID one-step RT-PCR kit” (Applied Biosystems, USA) and was carried out with previously described primers and probe [30]. Assays were run in duplicate and used a calibrated standard curve generated with serial dilutions (ranging from 10^1^ to 10^7^) of a plasmid clone previously characterized as genotype 3 [6]. Target copy numbers were calculated based on Ct values in reference to the standard curve. Lastly, the number of copies per millilitre was determined by adjusting the values according to the volumes that were used for each step of the procedure (i.e., extraction, and the qRT-PCR reaction).

### Detection of positive and negative strand RNA by nested RT-PCR

Serum, faecal and tissue samples collected at inoculum and at necropsy were tested by RT-PCR. In order to detect and characterize the HEV RNA positive-strand, two nested RT-PCR assays were carried out for amplification of partial fragments of 287 base pairs (bp) (ORF1) and 342 bp (ORF2), corresponding to methyltransferase and capsid genes of HEV, respectively. Complementary DNA (cDNA) synthesis and PCR amplifications were done in a single-tube using the SuperScript® III One-Step RT-PCR System with Platinum® Taq DNA Polymerase (Invitrogen Life Technology, CA, USA), in the presence of the specific external ORF1 and ORF2 primers, and carried out as previously described [31, 32]. Then, two sets of specific internal primer pairs were used in the nested step.

To further identify HEV replication sites, tissue samples that had detectable positive-strand HEV RNA were also tested for detection of negative-sense HEV RNA by both, ORF1 and ORF2 nested RT-PCR assays. The extracted RNA was reverse transcribed at 42°C for 60 min with the SuperScript III reverse transcription (Invitrogen Life Technology, CA, USA), in the presence of the external forward primer of both genome regions. Then, PCR and nested PCR were carried out to amplify the partial fragments of ORF1 and ORF2 as described above.

### Sequencing reactions and phylogenetic analyses

ORF1 and ORF2 products (amplified from the inoculum and liver samples of monkeys AE6, AB19 and AC7 obtained at necropsy) were purified using reagents and protocols of the QIAquick Gel Extraction kit (Qiagen) and sequenced using reagents and protocols of the Big Dye Terminator 3.1 kit. A Bayesian phylogenetic tree was constructed by using concatenated partial nucleotide sequence of ORF1 and ORF2 (546 bp) of HEV. Multiple nucleotide sequence alignment was analysed by using the Markov Chain Monte Carlo method implemented in the program MrBayes version 3.1.2 under GTR+G+I nucleotide substitution model, selected using the jModeltest program. The partial genomic sequences reported in this study have been deposited in the GenBank under accession numbers: inoculum ORF1/ORF2-KX578263/KX578267; liver AE6 ORF1/ORF2-KX578264/ KX578268; liver AB19 ORF1/ORF2-KX578265/ KX578269; and liver AC7 ORF1/ORF2-KX578266/KX578270.

### Biochemical and haematological analyses

Haematological analyses were carried out in the Sysmex XT-2000iv automated haematology analyzer (Sysmex Corporation, Kobe, Japan). Biochemical analyses were performed using a Vitros DT60 II chemistry system (Johnson & Johnson's, Minnesota, USA). The following parameters were evaluated: red and white blood cell counts (RBC and WBC), alanine aminotransferase (ALT), aspartate aminotransferase (AST), total bilirubin (TBIL), creatinine (CREA), blood urea nitrogen (BUN), glucose (GLU), albumin (ALB), and cholesterol (CHOL). Serum levels of glycated haemoglobin were determined by using the Dimension RxL Max integrated chemistry system (Siemens Healthineers, Muenchen Germany). Baseline levels were determined from blood samples of the pre-inoculation period for each animal. Biochemical evidence of hepatitis was considered when the post-inoculation serum levels of ALT exceed by > 2-fold the pre-inoculation baseline levels.

### Histopathological analyses

Liver tissues were obtained by ultrasound-guided biopsy, as described above, and at necropsy. A portion of each obtained sample was stored in 10% buffered formalin (pH 7.0). Paraffin sections from liver tissues were sectioned at 4 micrometre (μm) and stained with haematoxylin-eosin (Sigma-Aldrich, USA). Light photomicrographs were made in a DMRXA (Leica®) microscope mounted with a DFC300FX camera using Leica® and a Qwin Standard software. Liver sections were blindly examined and lesions were scored according to a previously classification for non-alcoholic fatty liver disease (NAFLD) by the non-alcoholic steatohepatitis Clinical Research Network (NASH CRN) [33].

### HEV antigen detection

Frozen liver sections (4 μm) obtained at 160 dpi were examined by indirect immunofluorescence using the Mouse Monoclonal (IgG1, 1 mg/mL) [clone 4B2] (LifeSpan BioSciences, Inc) that recognizes HEV ORF2 at a 1:150 dilution as the primary antibody. The Donkey Anti-Mouse IgG H&L (Alexa Fluor® 488; 2 mg/mL) (Abcam plc, Cambridge, UK) was used as the secondary antibody at a 1:1.500 dilution. The slides were mounted with SlowFade Gold Antifade Reagent with DAPI (Life Technologies Corporation, USA), and covered with a coverslip. Images of the positive fields were obtained by immunofluorescence microscope (Zeiss Axio Observer Z1, Göttingen, Germany).

### Bioequivalence of tacrolimus

The blood levels of tacrolimus in the cynomolgus monkeys were analysed by liquid chromatography (LC)—mass spectrometry system (LC-MS/MS). The method was validated according to the criteria of RDC27, 17/05/2012—Agência Nacional de Vigilância Sanitária (Anvisa). The tacrolimus reference standard was provided by United Stated Pharmacopeia (Rockville, MD, USA) and, diazepan used as the internal standard solution, was provided by National Institute for Quality Control in Health (INCQS/BRASIL) The LC-MS/MS was composed of liquid chromatography Prominence LC-20AB (Shimadzu, Kyoto, JP) and tandem mass spectrometry API 4000 (ABSCIEX, Foster City, CA, USA). The data were processed with the Analyst® software (version 1.6.2). The mass spectrometry was operated with electrospray ionization in the positive mode, and transitions at m/z 822.69 > 769.59 and m/z 284.99 > 192.77 were monitored for tacrolimus and internal standard, respectively. Nitrogen was used as the nebulizer and auxiliary gas.

### Statistical analyses

Two logistical models were applied based on HEV RNA detection on faeces (model 1) and sera (model 2). For each model, a previous univariate logistic regression was performed in order to select the variables for the multivariate model. The following variables were included: liver enzyme levels (AST, ALT, and TBIL), CREA, BUN, GLU, ALB, and CHOL levels, haematological parameters, immunosuppressive regimen (dose and bloody concentration), age, sex, body weight and temperature, and antibodies levels (IgM, IgG and IgA). Only the variables with significance of P < 0.40 were considered for the final logistic multivariate analysis and calculated the odds ratio for the final variables. All analysis were performed using the R Project for Statistical Computing (http://www.r-project.org/, and the software GraphPad Prism 5 for windows, version 5.01 (San Diego, CA, United States). For these models, we drop the control group (G3), since it was not exposed to HEV.

## Results

**Fig 1:**
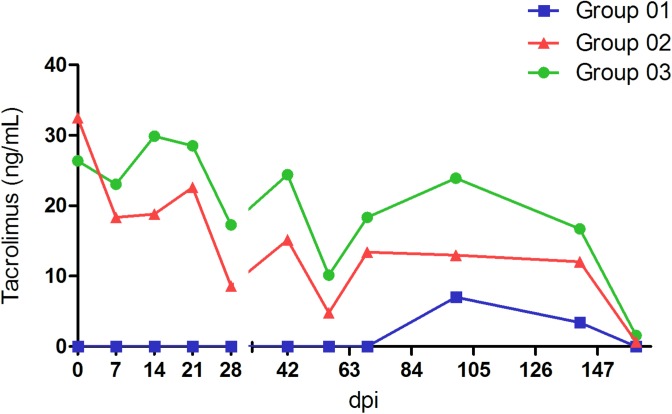
Liquid chromatography-mass spectrometry measurement of tacrolimus in blood samples from cynomolgus monkeys.

Both, G1 and G2 monkeys inoculated with swine HEV became infected. HEV infection was evidenced and monitored by HEV RNA in sera, faeces, and liver samples; anti-HEV antibodies; ALT and AST (Table 2); and histopathological features (S1 Table) throughout the experiment. The tacrolimus blood levels were monitored throughout the experiment (Fig 1).

**Table 2:** Summary of post infection detection of HEV RNA, seroconversion and aminotransferaases peaks in G1, G2, and G3 cynomolgus monkeys.

		HEV RNA detection dpi*			Anti-HEV seroconversion dpi*			Aminotransferases peaks** dpi* (corresponding values in IU/L)	
Monkey ID	Group	Serum	Faeces	Liver	IgA	IgM	IgG	ALT	AST
**AD4**	**1**	7–28	7–28	14–69	28	28	28	21, 28 (125, 173)	21 (127)
**AE3**		7–21	7–42	14	57	28	28	-	-
**V12**		14–21	7–28	14	57	28	28	21 (67)	21; 99 (97; 96)
**AC11**		14–21; 56	7–21	14	28	28	28	-	-
**AE6**	**2**	7–160	7–160	14–160	141	99	57	42; 56; 160 (82; 89; 84)	21 (93)
**AB19**		14–160	7–160	14–160	57	14	57	141; 160 (76; 120)	7; 14; 21; 42; 99 (106; 76; 84; 88; 95)
**AD8**		14–42	7–42	14–42	57	28	28	-	-
**AC7**		14–160	7–160	14–160	99	14	57	28; 42; 56; 99; 141; 160 (69; 122; 145; 71; 149; 90)	21; 42; 56; 69 (107; 74; 125; 80)
**AB17**	**3**	---	---	---	---	---	---	---	---
**AD7**		---	---	---	---	---	---	---	---
**Z2**		---	---	---	---	---	---	---	---
**AE2**		---	---	---	---	---	---	---	---

* **dpi**: days post inoculation

** **Aminotransferases peaks**: ALT/AST serum levels exceeding at least twice the individual baseline levels.

### Immunocompetent monkeys infected with HEV-3 (Group 1)

**Fig 2:**
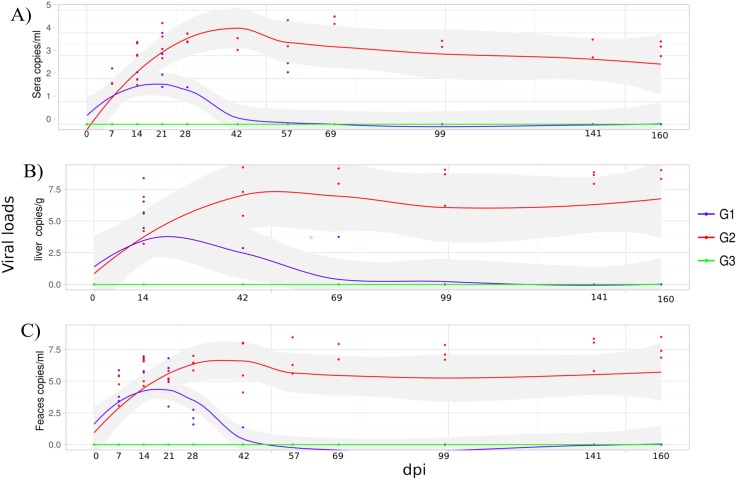
Dynamic of HEV RNA measured by quantitative reverse transcription PCR qRT-PCR. Immunocompetent monkeys (G1) were inoculated with HEV-3; tacrolimus-treated monkeys inoculated with HEV-3 (G2) and tacrolimus-treated monkeys (G3). qRT-PCR assay were performed on RNA extracted from sera, liver biopsies and faeces samples. The viral load results are show in: **(A)** sera, **(B)** liver biopsies, and **(C)** faeces.

HEV viremia (Table 2) was detected from 7–14 to 21–28 dpi excepting AC11, which exhibited an intermittent pattern of viremia at 56 dpi. Similar findings were noticed for viral faecal shedding excepting AE3, which remained excreting virus in faeces until 42 dpi. As expected, HEV RNA was detected in the liver for all monkeys at the first biopsy pi and remained detectable for at least 69 dpi in one of them (AD4). AD4 monkey was euthanized from an unrelated cause before the end of the study (71 dpi). The remaining monkeys from this group, AE3, V12 and AC11, showed viral clearance within 69 days or less (Fig 2A–2C). All monkeys seroconverted to anti-HEV IgM and IgG at 28 dpi and to anti-HEV IgA between 28–57 dpi. In two monkeys (AD4 and V12), a transient but significant elevation of ALT/AST levels was noticed at 21 dpi. The average number of platelets, RBC and WBC, and biochemical parameters did not change over the course of study.

**Fig 3:**
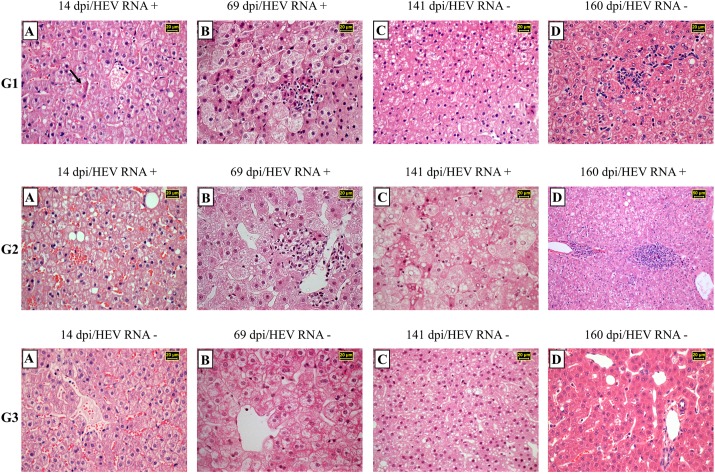
Chronological analysis of histopathological features of liver biopsies from immunocompetent cynomolgus monkeys infected with HEV (G1), monkeys previously treated with tacrolimus and infected with HEV (G2) and monkeys only treated with tacrolimus (G3). **Hematoxylin-eosin (H&E)–stained paraffin section. (G1A)** Lobular architecture disorganization, ballooning degeneration of the hepatocytes with lytic necrosis. Acidophilic clumps (arrow) are located in a perinuclear position (Mallory bodies). **(G1B)** The liver cytoarchitecture was modified by ballooning and lytic necrosis of hepatocytes. Lipid droplets and inflammatory cell infiltration were observed. **(G1C)** Hepatic parenchyma shows significantly fat droplets deposition, mixture of macrosteatosis and microsteatosis. **(G1D)** Focal collection of lymphocytes and macrophages localized in the pericentral area. Ballooned hepatocytes and lytic necrosis were noted. **(G2A)** Irregular distribution pattern of hepatocytes, ballooning ad cytolitic necrosis associated with fatty changes. **(G2B)** Normal liver architecture. Lymphohistiocytic infiltration of portal liver tract. Microvesicular steatosis is also present. **(G2C)** Disarray of the cytoarchitecture of the parenchyma. Hepatocytes exhibiting ballooning, lytic necrosis and steatosis. Note glycogen accumulation in hepatocyte nuclei. **(G2D)** Interface hepatitis surrounded by micro and macrosteatosis. **(G3A)** Normal hepatic venule. Hepatocytes plates shows regular distribution. **(G3B)** Lobular disarray and hepatocellular ballooning predominantly distributed in zone 3. **(G3C)** Significative and strong diffusely distributed mixture of hepatocellular macrosteatosis and microsteatosis in all zones from zone 1 to 3. **(G3D)** Hepatocytes cords converging towards portal tract. Minimal fat droplets deposition in the hepatocyte cytoplasm.

**Fig 4:**
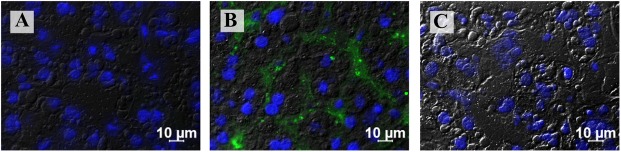
Immunofluorescence staining using monoclonal antibody to detect HEV antigen in the liver from cynomolgus monkeys obtained at the end of the experiment. **(A)** Negative immunofluorescence of monkey from G1 after viral clearance. **(B)** Detection of HEV antigen (labelled in green) in sinusoidal lining cells of chronically infected monkey from G2. **(C)** Negative results in control monkey from G3. Cell nuclei were counterstained with DAPI (blue).

The main liver histopathological findings at the acute phase of infection (at 14, 42 and 69 dpi) revealed mild to moderated macro and microvesicular steatosis (more evident in zone 1), hepatocellular ballooning degeneration (zone 3), intra-acinar inflammation, and mild intensity of hepatocellular apoptosis (Fig 3G1A and Fig 3G1B). Ballooning degeneration, mild to severe steatosis, apoptosis, and some inflammatory infiltrates were continuously diagnosed until 160 dpi (Fig 3G1C and Fig 3G1D). At the end of study, G1 animals neither showed evidence of chronic hepatitis, nor detectable HEV Ag (Fig 4A), or HEV RNA in liver sections. HEV infection in this group did not result in overt clinical signs.

In order to evaluate the possibility of reactivation of HEV infection, tacrolimus was administered (PO. 2.0 mg/kg/day) from 91 to 149 dpi to all animals of G1. G1 animals achieved a lower tacrolimus blood concentration, in comparison with G2 and G3 (Fig 1). Despite of small focus of acute inflammation detected in liver parenchyma at 160 dpi (Fig 3G1D), liver enzymes were not elevated. Neither HEV RNA nor HEV Ag were detected, even after tacrolimus challenge.

### Immunosuppressed monkeys infected with HEV-3 (G2)

**Fig 5:**
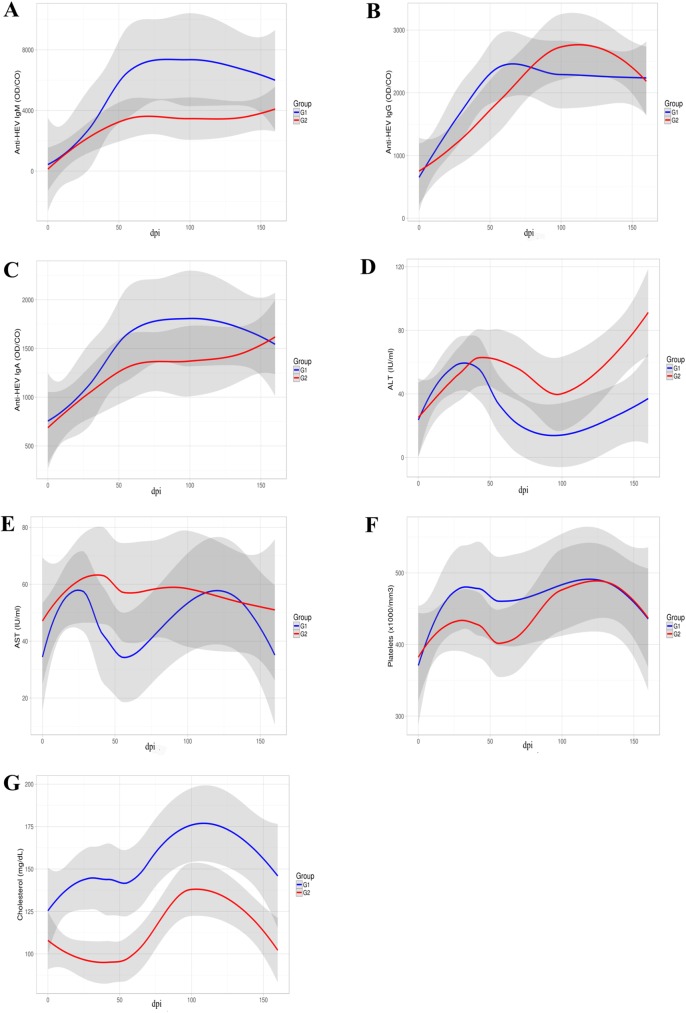
Distributions of anti-HEV antibodies, ALT, AST, platelets and CHOL observed in inoculated groups (G1 and G2). Anti-HEV IgM, IgG and IgA are shown in **A**, **B**, and **C**, respectively. Samples with OD/cutoff ratios above 1.0 are considered positive for anti-HEV. ALT and AST levels are show in **D** and **E**, and platelets and cholesterol levels in **F** and **G**.

In this group, the blood tacrolimus concentrations ranged from 4.78 ng/ml to 18.83 ng/ml (Fig 1). In respect of viral load in blood, liver, and faecal samples at the acute stage of infection, G2 and G1 monkeys showed similar patterns. After three months of infection, three out of four HEV inoculated monkeys (AE6, AB19, and AC7) revealed a persistent pattern of viremia, faecal shedding and presence of HEV RNA in all liver biopsies compatible with a chronic HEV infection (Fig 2A–2C). The exception AD8, exhibited viral clearance within 69 dpi, similar to that observed in G1 (Table 2). Monkeys from G2 showed lower IgM titres in comparison with immunocompetent animals, even though no significant difference was reported at multivariate analysis (Fig 5A). The appearance of anti-HEV-IgG antibodies was a little later, as well as its peak (Fig 5B). Moreover, the three chronically HEV infected monkeys exhibited an intermittent pattern of ALT and AST elevated levels (Fig 5D and Fig 5E, Table 2). Again, monkey AD8 did not show changes in liver enzymes levels. As observed in G1, during the experiment, no variation of the average number of platelets, RBC, WBC, and other biochemical parameters was noticed. However, the counting of platelets numbers in G2 was slightly lower to the observed in G1 in the first 60 dpi, but no significant statistical difference was noted (Fig 5F). A significant lower level of CHOL was noted in G2 comparing to G1 as we can observe in Fig 5G.

At 14 and 42 dpi, challenged monkeys exhibited few ballooning degeneration, mild steatosis (Fig 3G2A), scattered hepatocellular apoptosis, and lobular inflammation. At 69 dpi, all monkeys revealed mild to severe macro and microsteatosis (zone 1), marked ballooning degeneration (zone 3), few hepatocellular apoptosis, and at most mild portal and lobular inflammations (Fig 3G2B). At the chronic stage (160 dpi), a limited area of interface hepatitis (piecemeal necrosis) (Fig 3G2D) was observed in the tacrolimus treated animals, with the exception of the AD8 monkey, that presented a self-limited acute hepatitis characterized by absence of inflammatory cell infiltrate in the liver parenchyma. In addition, HEV antigens were observed in the liver sections of all monkeys (Fig 4B), with exception of AD8.

**Fig 6:**
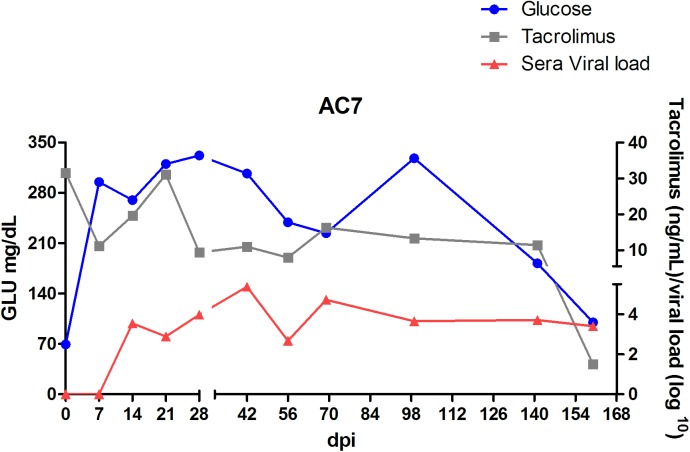
Tacrolimus concentrations measured by LC-MS/MS in whole blood, HEV viral load and blood glucose level in AC7 monkey previously treated with tacrolimus and infected with HEV.

All animals from this group presented alopecia in dorsal left and right forelimbs and hind limbs (S1 Fig), and moderate weight loss throughout the experiment. AC7 and AB19 showed herpetic lesions (S2 Fig) confirmed by PCR on oral mucosal and lips. Moreover, AC7, who presented dermatitis, polyuria and polydipsia, developed diabetes mellitus with a markedly elevated level of blood glucose, ranging from 181 to 327 mg/dl through the experiment and glycated haemoglobin (8.8% at 78 dpi). Hyperglycaemia was first reported 49 days after the beginning of the tacrolimus treatment (at 7 dpi) (Fig 6). At the end of the experiment, glycaemic levels start declining to normal levels. Fig 3G2C shows a massive presence of glycogen accumulation in the nuclei of hepatocytes, an indicative of diabetes mellitus.

**Fig 7:**
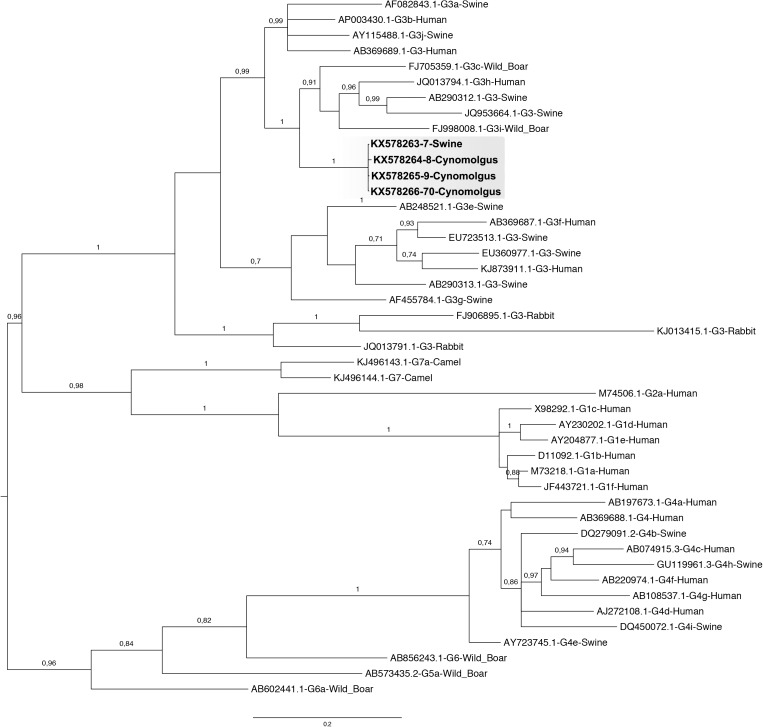
Phylogenetic analysis of Hepatitis E virus strains from human and animal samples. The Bayesian phylogenetic tree was constructed by using concatenated partial nucleotide sequence of ORF1 and ORF2 (546 nt) of HEV. For each sequence used, the GenBank accession number, animal species from which it was isolated and the corresponding genotype are shown. The tree was rooted at midpoint. Posterior probabilities (pp) are shown at the branch label. Numerical value ≥ 0.7 indicates the pp replicates that supported the interior branch. Newly described HEV sequences in this study are indicated. Scale bar indicates evolutionary distance of 0.2 substitutions per position in the sequence.

Both positive- and negative-strand HEV RNA, were detected in hepatic (liver and gallbladder) and extrahepatic tissues (spleen, duodenum, colon, lymph node and pancreas) taken from the persistently infected monkeys AE6, AB19 and AC7. Bile, jejunum and ileum also presented the positive but not the negative-strand RNA, whereas, liquor, thymus and brain were negative for viral detection. No viral RNA was detected in tissue samples from AD8. As determined by direct sequencing, sequences found in liver samples from the three chronic infected animals matched with the original inoculum. Phylogenetic reconstruction using concatenated partial nucleotide sequences of ORF1 and ORF2 (546 nt) clustered all the samples within genotype 3, clade abchij, subclade chi (Fig 7).

### Comparison between immunocompetent (G1) and immunosuppressed (G2) HEV infected groups

**Fig 8:**
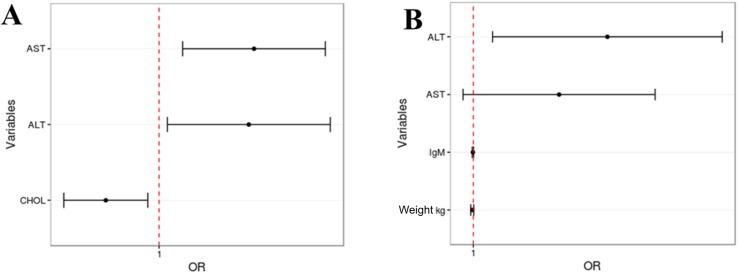
Predictive factors associated with chronic HEV infection. **(A)** Predictive factors obtained based on faeces viral load. **(B)** Predictive factors obtained based on sera viral load.

Univariate and multivariate analysis models were performed to identify variables associated with chronic HEV infection. In model 1, univariate analysis pointed the following variables: age, sex, weight, IgM, IgG, IgA, tacrolimus dose, ALT, AST, TBIL, GLU, CREA, CHOL, BUN. After adjustments for model 1, the multivariate analysis revealed the following independent predictive factors associated with chronic HEV infection in G2: ALT (odds ratio = 1.0432; 95% confidence interval, 1.00392–1.0840; P = 0.030781), and AST (odds ratio = 1.0457; 95% confidence interval, 1.01120–1.0815; P = 0.009062) as risk factors; and CHOL appears as protection factor (odds ratio = 0.9752; 95% confidence interval, 0.95612–0.9947; P = 0.012968) (Fig 8A). In model 2, univariate analysis pointed the flowing variables: age, weight, IgM, IgA, tacrolimus dose, ALT, AST, TBIL, GLU, CREA, and CHOL. After adjustments for model 2, variables IgM, ALT, AST, and weight remain in the final model, but only ALT was considered a risk factor for chronic HEV infection (odds ratio = 1.05453; 95% confidence interval, 1.007643–1.1036; P = 0.02213) whereas IgM (odds ratio = 0.99983; 95% confidence interval, 1.0001–0.999567; P = 0.999567), AST (odds ratio = 1.03456; 95% confidence interval, 1.0747–0.995963; P = 0.07987), and weight (odds ratio = 0.99959; 95% confidence interval, 1.0002–0.998998; P = 0.16941) were not (Fig 8B).

### Immunosuppressed monkeys not infected (G3)

In the control group, the blood tacrolimus concentrations ranged from 10.13 ng/ml to 29.89 ng/ml (Fig 1). All animals presented normal ALT/AST levels, and were negative for anti-HEV antibodies, HEV RNA (faeces, sera, and liver biopsies) throughout the experiment and at necropsy. No haematological and biochemical variation was observed in this group.

Histologic features revealed hepatocellular ballooning degeneration, hepatocytes binucleation and cytolitic necrosis (Fig 3G3A–3G3D). HEV antigen was not detected in liver samples from any monkey (Fig 4C).

Similarly to G2, all animals presented moderate loss weight and bilateral alopecia in the same body regions. Monkeys AD7 and Z2 got severely ill by opportunistic herpes virus infection, and they were euthanized at 45 and 71 dpi, respectively.

## Discussion

Recently, autochthonous HEV infection has emerged as an important cause of morbidity among immunocompromised patients, since it can progress to chronic hepatitis in approximately 60% of transplant recipients who are exposed to HEV [19, 20]. The risk is increased in patients receiving tacrolimus therapy at the time of hepatitis E diagnosis [19]. Both, the risk of reactivation and the incidence of a de novo HEV infection after solid organ transplantation have been reported, in Germany, the Netherlands and France [19, 34, 35]. So far, HEV infection in Brazil is similar to that observed in developed countries, with HEV-3 being detected in humans and pigs [3, 6, 36]. Indeed, few indigenous cases of human hepatitis E have been described in Brazil, mostly in SOT immunosuppressed patients, all classified within genotype 3 [4, 5, 36]. The present study describes a successful experimental chronic infection of immunosuppressed cynomolgus monkeys with a Brazilian swine HEV-3 isolated from a naturally infected pig.

In our study both, tacrolimus-treated and -untreated NHP groups inoculated with the Brazilian swine HEV-3 developed subclinical hepatitis. However, the pattern of infection in the untreated group (G1) varied somewhat, as previously reported, in experimental studies using immunocompetent NHP [25, 37]. The first sign of HEV infection was the viral shedding in faeces within 7 dpi, followed by seroconversion to anti-HEV IgM and IgG within 28 dpi, discrete liver enzymes elevation within 21–28 dpi, associated with a mild liver inflammation. Lastly, viral clearance was achieved spontaneously in this group within 69 dpi or earlier. In immunocompetent patients infected with HEV-3, clinical symptoms occur in only 2–5% [38]. Here, the subclinical hepatitis noted in our untreated NHP reflects a similar scenario

Chronic human HEV infection is defined as persistently HEV replication longer than three months, with mild elevation of liver enzymes, and infrequent association with clinical signs [18]. In our study, the immunosuppressed NHP AE6, AB19 and AC7 became chronically infected, as hypothesized. As observed in SOT receptors [20], the present study showed that HEV viral loads (in sera, faeces or liver) and anti-HEV IgM/IgG levels obtained during acute infection phase were not able to predict the progression to chronic hepatitis E. Previous studies reported that serological tests may give false negative results in SOT patients, so that HEV antibodies are infrequently detected at the acute phase [20, 34, 39]. Besides, the detection of IgG and IgM anti-HEV may be delayed in patients treated with immunosuppressants including calcineurin inhibitors [39]. In our experiment, all chronically infected monkeys seroconverted to anti-HEV antibodies, however, those that developed chronic infection showed a delayed seroconversion. In addition, tacrolimus-treated NHP showed lower IgM and IgA titres throughout the experiment when compared with untreated NHP, even though no significant difference was observed.

Intriguingly, notwithstanding tacrolimus treatment, one out of four of the immunosuppressed NHP (AD8) developed a self-limited acute hepatitis—characterized by absence of inflammatory cell infiltrates in liver parenchyma, poor liver enzymes elevation, spontaneous viral clearance, and HEV-specific seroconversion. Similarly, 40% of the HEV infected immunosuppressed SOT patients are able to clear the virus [19]. The activation of the innate immune response and the development of a multispecific CD4 + and CD8 + T-cell response to ORF2 seem to be critical for the clearance of the virus in patients with hepatitis E [40]. Besides, a weak inflammatory response, and high serum concentrations of chemokines in the acute phase of hepatitis E have been associated with the persistence of HEV infection [41]. Therefore, self-limited acute outcome of HEV infection has been associated with a potent and multispecific T-cell response.

In our study, the animals that developed chronic HEV infection had low peak transaminases levels (ALT and AST); the same has been observed in SOT patients who evolved to chronic infection [19]. As previously demonstrated, at acute phase, liver enzymes levels are higher in self-limited HEV infections [20]. Nevertheless, the tacrolimus-treated monkey who spontaneously resolved infection did not show liver enzymes changes. In the present study, persistently increased ALT and AST levels were predictors of progression to chronic HEV infection, as observed in HBV chronically infected patients [42].

Cyclosporine, which is also a calcineurin inhibitor, has been frequently associated with hyperlipidemia in SOT receptors [43]. In contrast, in our study reduced plasma levels of cholesterol were significantly associated with chronic HEV infection in the tacrolimus-treated group. It can be explained by the evident abnormal fatty deposit (macro and micro steatosis) in liver parenchyma [44], as observed in our study (G2 animals). Regarding haematological features, the WBC count was not reduced by tacrolimus treatment, as expected [45] and platelets account could not be associated with HEV chronicity, as previously reported in SOT patients infected with HEV [19]. Mild to moderate thrombocytopenia after transplantation can be observed due to many factors, such as reduced hepatic thrombopoietin production, allograft sequestration, hypersplenism, haemorrhage, heparin-induced thrombocytopenia, immunologic reactions, hemolysis, drugs, infections, and sepsis [46, 47]. However, in the present study, the animal model was not submitted to the surgical procedures that could result in mild to moderate thrombocytopenia in SOT patients. Both, drug-induced and immune-mediated (severe) thrombocytopenia has been associated with the use of tacrolimus [48] and due to HEV infection, respectively [49, 50]. In our study, all the infected animals showed platelet counts within the normal range at the time of infection and throughout the experiment. Still and all, three out four tacrolimus-treated monkeys became persistently infected by HEV-3.

Tacrolimus blood levels above 10 ng/ml have been used successfully to prevent renal allograft rejection in cynomolgus monkeys [27, 28]. The rate of absorption and the bioavailability of tacrolimus after oral administration (which was the chosen administration via, in our study) appear to be variable in all the patient populations studied [51]. It was extremely difficult to control the blood concentrations of oral tacrolimus in our cynomolgus monkeys, as also reported by other authors [52], therefore the dose had to be adjusted throughout the experiment. At 149 dpi, tacrolimus dose was reduced from 3.5 mg/kg/day to 2.0 mg/kg/day, since some animals exhibited herpetic lesions assumed as opportunistic infection. Besides that, G1 animals achieved a lower tacrolimus blood concentration, in comparison with G2 and G3. This fact may have interfered with our hypothesis of HEV reactivation.

Chronic HEV infection rapidly progress to fibrosis in 60% of SOT patients, and within 2 or 3 years 10% of these can become cirrhotic [18, 19, 53]. Therefore, chronic hepatitis E is typically more aggressive than chronic hepatitis B or hepatitis C [54]. In our study, chronic hepatitis was diagnosed according to typical histological features, such as interface hepatitis, at 160 dpi, in chronically infected monkeys. Liver fibrosis was not observed, probably due to the limited period of the study. Similar results—lymphocytic portal infiltrates with piecemeal necrosis (interface hepatitis)—were observed in heart and liver-transplanted recipients showing strong interface activity [13, 34, 39].

Previous studies have shown that HEV can replicate in organs and tissues other than the liver [55, 56]. Acute or chronic HEV infection may also cause extrahepatic manifestations that includes neurological disorders, kidney injury, acute pancreatitis and haematological abnormalities [57]. HEV RNA negative strand, which is indicative of replication, was detected in the liver, gallbladder, spleen, duodenum, colon, lymph node and pancreas from the three chronic infected monkeys. Recently, different HEV *quasispecies* were identified from serum and liquor from the same patient with chronic HEV, suggesting that chronic infection might promote the emergence of neurotropic variants of HEV [58]. In the short time of this study, it was not possible to detect HEV RNA in central nervous system (CNS), in contrast to that described in chronically infected rabbits [59]. Since histopathological changes were not observed in kidney tissues (data not shown), and no renal function changes were noticed, HEV RNA was not tested.

Common side effects of tacrolimus medication include nephrotoxicity, neurotoxicity, hepatotoxicity, and new-onset diabetes mellitus after transplantation (NOADT) [21, 60]. In order to assess the possible toxic effects of tacrolimus, we opted to include a control group (G3) of tacrolimus-treated-not-infected monkeys. Nevertheless, none animal from this group showed signs of neurotoxicity, nephrotoxicity or NOADT. The former is a clinical condition commonly observed in humans, and recently, was reported in an experimental study using tacrolimus-treated cynomolgus [27]. In our study one chronically HEV infected monkey, AC7, developed diabetes mellitus type 2 under immunosuppressive therapy, when daily blood levels of tacrolimus ranged 6.5–30 ng/ml and after HEV inoculation. Hyperglycaemia was first noticed (294 mg/dl) at 49 days of the immunosuppressive treatment, one week pi and reached elevated levels (327 mg/dl) associated to increased levels of glycated haemoglobin (8.8%) throughout the experiment. Glycaemia turned into the normal level (99 mg/dl) as soon as tacrolimus treatment was discontinued, even when HEV infection was still active. These findings were similar to those described in heart-and-thymus-transplanted cynomolgus, submitted to a daily intramuscular immunosuppressive therapy including tacrolimus at blood concentrations of 20–30 ng/ml [27].

The relationship between calcineurin inhibitors, such as tacrolimus and cyclosporin, and the development of NOADT is widely recognized in human medicine. Patients on cyclosporine treatment has been shown lower risk of developing NOADT than those using tacrolimus [61]. The mechanism of NOADT is multifactorial with high incidence in the first year after transplantation and immunosuppressive treatment. Besides exposure to pharmacologic agents, other events like hepatitis C infection in SOT recipients also confer an increased risk of NOADT development [61, 62]. However, no association between chronic HEV infection in immunosuppressed patients and NOADT development was reported until now.

In conclusion, this study has provided the evidence that immunosuppressed cynomolgus monkeys treated with tacrolimus and infected with swine HEV-3 can develop chronic HEV infection. HEV infection was associated with interface hepatitis, characterizing the beginning of a chronic hepatitis. Furthermore, the cross-species transmission reported here reinforces the zoonotic role of swine HEV-3 strains in epidemiology of HEV infection in Brazil.

## Supporting information

S1 Fig Clinical appearance of alopecia in fore limbs and hind limbs, with well-circumscribed areas of hair loss without underlying skin inflammation. (TIF)

S2 Fig Lesion caused by herpetic reactivation. (TIF)

S1 Table Time course analysis of histopathological changes of liver biopsies. **(A)** analysis from immunocompetent monkeys infected with HEV (G1); **(B)** analysis from monkeys previously treated with tacrolimus and infected with HEV (G2); and **(C)** analysis from monkeys only treated with tacrolimus (G3).
(DOCX)
