# Supplementary figures and images for: Cynomolgus monkeys are successfully and persistently infected with hepatitis E virus genotype 3 (HEV-3) after long-term immunosuppressive therapy
Source: PLoS One. 2017 Mar 22;12(3):e0174070. doi: 10.1371/journal.pone.0174070 (PMC5362194; doi:10.1371/journal.pone.0174070)

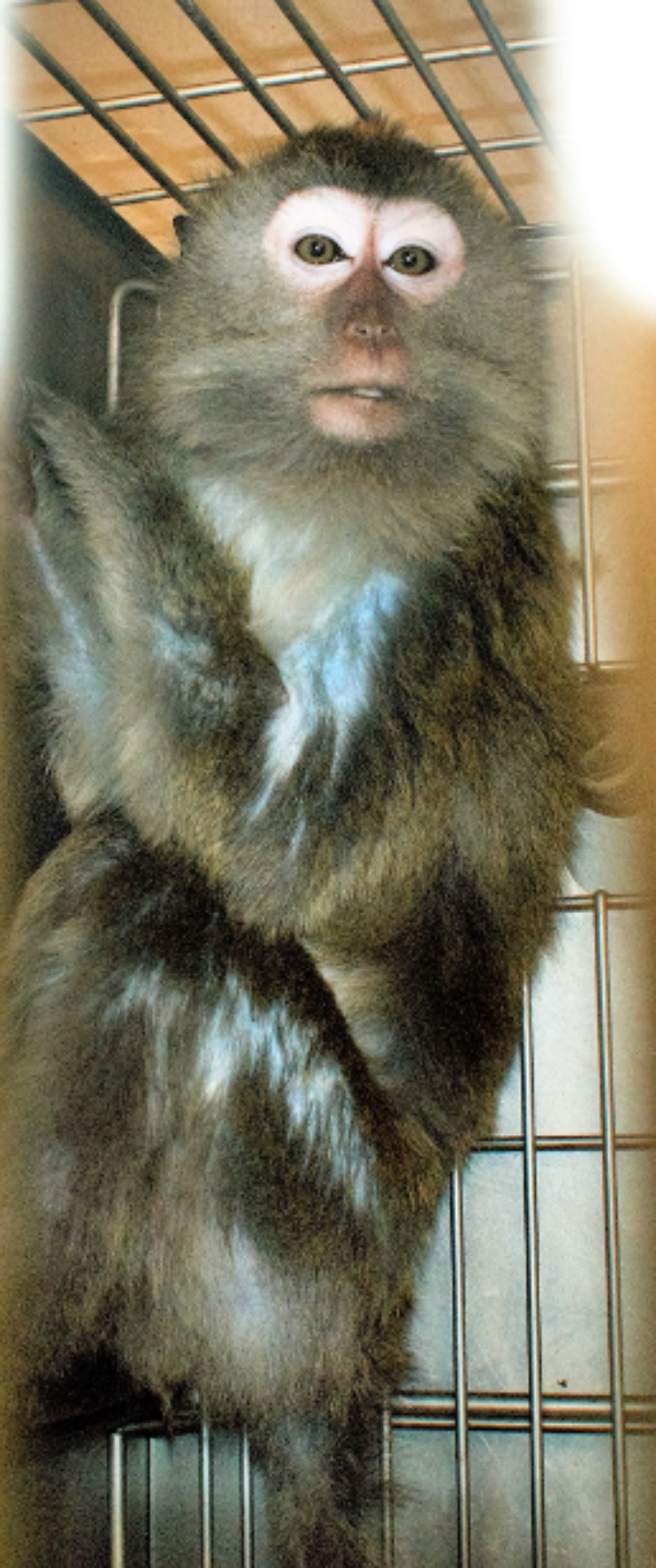

Supplement: S1 Fig — (TIF) [file pone.0174070.s001.tif]

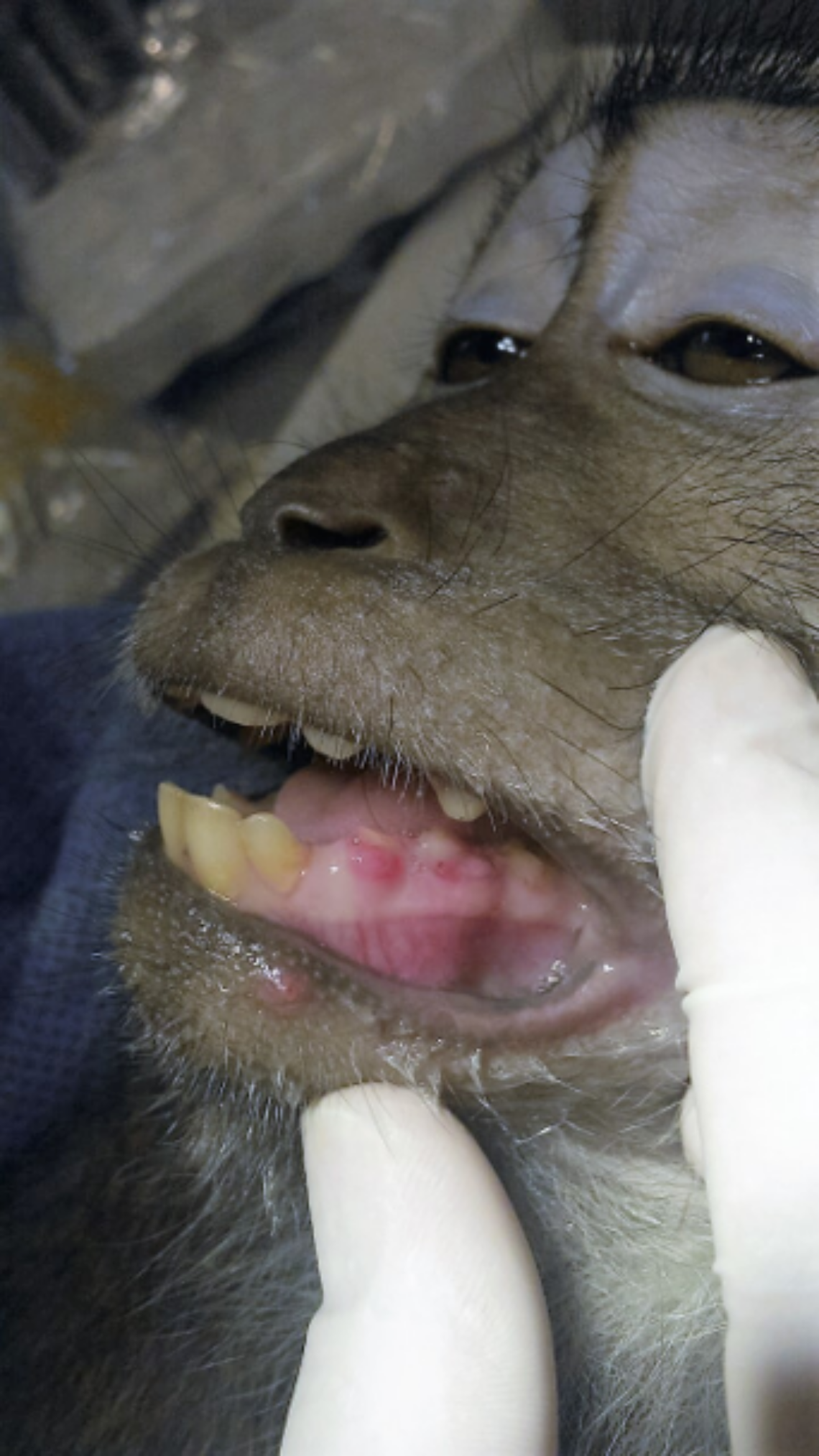

Supplement: S2 Fig — (TIF) [file pone.0174070.s002.tif]
