# Supplementary material for: Cynomolgus monkeys are successfully and persistently infected with hepatitis E virus genotype 3 (HEV-3) after long-term immunosuppressive therapy
Source: PLoS One. 2017 Mar 22;12(3):e0174070. doi: 10.1371/journal.pone.0174070 (PMC5362194; doi:10.1371/journal.pone.0174070)
Supplement: S1 Table — (A) analysis from immunocompetent monkeys infected with HEV (G1); (B) analysis from monkeys previously treated with tacrolimus and infected with HEV (G2); and (C) analysis from monkeys only treated with tacrolimus (G3). (DOCX) [file pone.0174070.s003.docx]

**(A)**

| **Histopathological findings** | 17/06/2014 | 16/09/2014 | 14/10/2014 | 10/11/2014 | 10/12/2014 | 21/01/2015 | 10/02/2015 |
| --- | --- | --- | --- | --- | --- | --- | --- |
|  | **0 dpi** | **14 dpi** | **42 dpi** | **69 dpi** | **99 dpi** | **141 dpi** | **160 dpi** |
| Steatosis **(0,1,2,3)** | 02/03 **(1)** | 03/04 **(0-2)** | 02/03 **(0-2)** | 03/04 **(0-2)** | 03/03 **(1-2)** | 03/03 **(2-3)** | 02/03 **(0-3)** |
| Hepatocellular ballooning **(0,1,2)** | 0/03 | 01/04 **(0-1)** | 0/03 | 03/04 **(0-2)** | 02/03 **(0-2)** | 01/03 **(0-1)** | 0/03 |
| Fibrosis **(0,1,2,3,4)** | 0/03 | 0/04 | 0/03 | 0/04 | 0/03 | 0/03 | 0/03 |
| Lobular inflammation **(0,1,2,3)** | 0/03 | 0/04 | 0/03 | 0/04 | 0/03 | 0/03 | 01/03 **(0-1)** |
| Portal tract inflammation **(0,1,2,3)** | 0/03 | 0/04 | 0/03 | 0/04 | 0/03 | 0/03 | 01/03 **(0-1)** |
| Interface hepatitis | 0/03 | 0/04 | 0/03 | 0/04 | 0/03 | 0/03 | 01/03 **(0-1)** |
| Apoptosis **(0,1,2)** | 0/03 | 0/04 | 01/03 **(0-1)** | 01/04 **(0-2)** | 02/03 **(0-1)** | 01/03 **(0-1)** | 0/03 |
| Lytic necrosis | 0/03 | 01/04 **(1)** | 01/03 **(1)** | 0/04 | 0/03 | 01/03 **(1)** | 0/03 |
| Focal necrosis | 0/02 | 0/04 | 0/03 | 01/03 **(0-2)** | 0/03 | 0/03 | 0/03 |
| Mallory-Denk bodies | 0/02 | 01/04 | 0/03 | 0/3 | 0/03 | 0/03 | 0/03 |

**(B)**

| **Histopathological findings** | 17/06/2014 | 16/09/2014 | 14/10/2014 | 10/11/2014 | 10/12/2014 | 21/01/2015 | 10/02/2015 |
| --- | --- | --- | --- | --- | --- | --- | --- |
|  | **0 dpi** | **14 dpi** | **42 dpi** | **69 dpi** | **99 dpi** | **141dpi** | **160 dpi** |
| Steatosis **(0,1,2,3)** | 04/04 **(0)** | 02/03 **(0-1)** | 01/02 **(0-1)** | 04/04 **(1-3)** | 04/04 **(1-3)** | 04/04 **(2-3)** | 04/04 **(1-3)** |
| Hepatocellular ballooning **(0,1,2)** | 01/04 **(0-1)** | 02/03 **(0-1)** | 01/02 **(0-1)** | 03/04 **(0-2)** | 04/04 **(1-2)** | 04/04 **(1-2)** | 04/04 **(1-2)** |
| Fibrosis **(0,1,2,3,4)** | 0/04 | 0/03 | 0/02 | 0/04 | 0/04 | 0/04 | 0/04 |
| Lobular inflammation **(0,1,2,3)** | 0/04 | 0/03 | 0/02 | 01/04 | 0/04 | 0/04 | 01/04 **(0-1)** |
| Portal tract inflammation **(0,1,2,3)** | 0/04 | 0/03 | 0/02 | 0/04 | 01/04 **(2)** | 0/04 | 01/04 **(0-2)** |
| Interface hepatitis | 0/04 | 0/03 | 0/02 | 0/04 | 0/04 | 0/04 | 03/04 **(0-1)** |
| Apoptosis **(0,1,2)** | 0/04 | 0/03 | 01/02 **(0-1)** | 01/04 **(0-2)** | 01/04 **(0-1)** | 01/04 **(0-2)** | 0/04 |
| Lytic necrosis | 0/04 | 0/03 | 0/02 | 0/04 | 01/04 **(1)** | 01/04 **(1)** | 02/04 **(1)** |
| Focal necrosis | 0/04 | 0/03 | 01/02 **(1)** | 01/04 **(1)** | 01/04 **(1)** | 0/04 | 02/04 **(1)** |
| Mallory-Denk bodies | 0/04 | 0/03 | 0/03 | 0/04 | 0/04 | 0/04 | 0/04 |

**(C)**

| **Histopathological findings** | 17/06/2014 | 16/09/2014 | 14/10/2014 | 10/11/2014 | 10/12/2014 | 21/01/2015 | 10/02/2015 |
| --- | --- | --- | --- | --- | --- | --- | --- |
|  | **0 dpi** | **14 dpi** | **42 dpi** | **69 dpi** | **99 dpi** | **141dpi** | **160 dpi** |
| Steatosis **(0,1,2,3)** | 2/04 **(0-1)** | 01/04 **(0-2)** | 02/04 **(0-2)** | 02/03 **(0-2)** | 01/02 **(0-2)** | 02/02 **(2-3)** | 02/02 **(1-3)** |
| Hepatocellular ballooning **(0,1,2)** | 0/04 | 02/04 **(0-2)** | 01/04 **(0-1)** | 03/03 **(1-3)** | 01/02 **(0-1)** | 01/02 **(0-1)** | 0/02 |
| Fibrosis **(0,1,2,3,4)** | 0/04 | 0/04 | 0/04 | 0/03 | 0/02 | 0/02 | 0/02 |
| Lobular inflammation **(0,1,2,3)** | 0/04 | 0/04 | 0/04 | 0/03 | 0/02 | 0/02 | 0/02 |
| Portal tract inflammation **(0,1,2,3)** | 0/04 | 0/04 | 0/04 | 01/03 **(0-1)** | 0/02 | 01/02 **(0-1)** | 0/02 |
| Interface hepatitis | 0/04 | 0/04 | 0/04 | 0/03 | 0/02 | 0/02 | 0/02 |
| Apoptosis **(0,1,2)** | 0/04 | 01/04 **(0-1)** | 0/04 | 0/03 | 0/02 | 0/02 | 0/02 |
| Lytic necrosis | 0/04 | 02/04 **(2)** | 0/04 | 02/03 **(1 e 2)** | 01/02 | 0/02 | 0/02 |
| Focal necrosis | 0/04 | 0/04 | 0/04 | 0/03 | 0/02 | 0/02 | 0/02 |
| Mallory-Denk bodies | 0/04 | 04/04 | 0/04 | 0/03 | 0/02 | 0/02 | 0/02 |
